# Complications Beyond Borders: A Case Study on a Retained Surgical Towel Leading to an Intra-abdominal Abscess Post-hysterectomy

**DOI:** 10.7759/cureus.72601

**Published:** 2024-10-29

**Authors:** Dallin Judd, Jaden Hawes, Jake Oldham, Brenton Stucki, Bryant Boyack DO

**Affiliations:** 1 Medicine, Texas College of Osteopathic Medicine, University of North Texas Health Science Center, Fort Worth, USA; 2 Emergency Medicine, Banner Baywood Medical Center, Mesa, USA

**Keywords:** intra-abdominal abscess, medical tourism, postoperative complications, retained foreign body, surgical safety

## Abstract

This report discusses a significant complication following a hysterectomy performed in Mexico on a 46-year-old woman who developed an intra-abdominal abscess due to a retained surgical towel - a severe but rare surgical error. Initially, her post-surgical symptoms, such as abdominal pain, fever, and malaise, were dismissed by her surgeon in Mexico as typical recovery effects. However, as her condition worsened, she sought emergency care in Arizona. A CT scan revealed a substantial abscess in her abdomen, identified by a radiodense ribbon indicating the forgotten surgical towel. Emergency surgery was required to remove the towel and treat the abscess, further complicated by the presence of a multi-drug-resistant organism.

This case underscores the importance of strict adherence to surgical safety protocols, the risks of seeking medical procedures abroad, and the critical need for thorough postoperative monitoring and clear communication between patients and healthcare providers.

## Introduction

Over 600,000 hysterectomy procedures are performed annually in the United States, making it the most common non-obstetric surgical procedure among women [1]. The advancement of this surgical procedure has improved the quality and duration of the lives of countless women worldwide. However, with surgical procedures, there is always a risk of severe complications, including bleeding, infection, damage to surrounding organs, and even mortality. Driven by economic considerations, many people within the United States resort to medical tourism to undergo surgical procedures in other countries despite the often higher incidences of complications. This case report follows the outcome of a middle-aged female from the United States who traveled to Mexico for a hysterectomy, wherein she developed an intra-abdominal abscess due to a retained surgical towel. This article was previously presented as a meeting abstract at the University of North Texas Health Science Center (UNTHSC) Research Appreciation Day on March 28, 2024.

## Case presentation

A 46-year-old female presented to her gynecologist in the United States with the primary complaint of abnormal vaginal bleeding. After consultation, the patient opted to proceed with a recommended hysterectomy. Due to difficulties scheduling the procedure, the patient chose to have the procedure performed in Mexico, where availability was more flexible and healthcare service costs were more affordable. A partial hysterectomy was performed in a hospital in Mexico without apparent complications. Subsequently, the patient was discharged from the facility and returned to her home in Arizona five days postoperatively.

On postoperative day 7, the patient began to experience worsening abdominal pain, fever, chills, and general malaise. The patient communicated her symptoms to her surgeon in Mexico and was told this response was normal following surgery. When symptoms had not improved 10 days after the surgery, she presented to the emergency room (ER) at a local hospital in Arizona. On physical examination, the patient exhibited tenderness in all four abdominal quadrants and guarding in the lower abdomen. Laboratory investigations revealed an elevated white blood cell count (WBC) of 16.0 × 10^9^/L and a marked elevation in the erythrocyte sedimentation rate, as demonstrated in Tables 1-2. A urinalysis was performed, revealing the presence of small amounts of blood and elevated protein levels (30 mg/dL) (Table 3). Blood gases were also assessed, showing a pH of 7.36 and a partial pressure of carbon dioxide (PCO₂) of 45 mmHg (Table 4).

**Table 1:** Complete blood count (CBC) obtained on postoperative day 10 Hgb: hemoglobin; Hct: hematocrit; MCV: mean corpuscular volume; MCH: mean corpuscular hemoglobin; MCHC: mean corpuscular hemoglobin concentration; Plt: platelets; RDW %: red cell distribution width percentage; MPV: mean platelet volume; GRAN: granulocyte; LYMPH: lymphocyte; MID: mid cells

Lab name	Lab value		Reference range
WBC	16.7 (H)	4.5-11.0 x 10³/µL	
RBC	3.1 (L)	4.7-6.1 M/µL (males), 4.2-5.4 M/µL (females)	
Hgb	9.4 (L)	13.8-17.2 g/dL (males), 12.1-15.1 g/dL (females)	
Hct	26.5 (L)	40-52% (males), 36-48% (females)	
MCV	84.5	80-100 fL	
MCH	30.2	27-33 pg/cell	
MCHC	35.7	32-36 g/dL	
Plt	648 (H)	150-450 x 10³/µL	
RDW %	12.6	11.5-14.5%	
PLT	648 (H)	150-450 x 10³/µL	
MPV	7.3 (L)	7.5-12.0 fL	
GRAN %	88.9 (H)	40-70%	
LYMPH %	8.5 (L)	20-40%	
MID %	2.6	2-8%	
GRAN ABS	14.8 (H)	1.5-8.0 x 10³/µL	
LYMPH ABS	1.4	1.0-4.0 x 10³/µL	
MID ABS	0.5	0.2-1.0 x 10³/µL	

**Table 2:** General chemistry labs obtained on postoperative day 10 BUN: blood urea nitrogen; ALT: alanine aminotransferase; AST: aspartate aminotransferase; ESR: erythrocyte sedimentation rate; eGFRer: estimated glomerular filtration rate; A/G: albumin/globulin

Lab name	Lab value		Reference range
Sodium	136	135-145 mEq/L	
Potassium	4.2	3.5-5.0 mEq/L	
Chloride	106	96-106 mEq/L	
CO_2_	22	22-28 mEq/L	
Anion gap	11	8-16 mmol/L	
Glucose level	92	70-99 mg/dL	
BUN	7	7-20 mg/dL	
Creatinine	0.68	0.6-1.2 mg/dL (males), 0.5-1.1 mg/dL (females)	
eGFRer	109	≥90 mL/min/1.73 m²	
Calcium	7.9 (L)	8.6-10.2 mg/dL	
Mg	2.1	1.7-2.2 mg/dL	
Protein, total	8	6.0-8.3 g/dL	
Albumin	2.6 (L)	3.5-5.0 g/dL	
Globulin	5.1 (H)	2.0-3.5 g/dL	
A/G ratio	0.4 (L)	1.0-2.2	
Bili total	0.4	0.1-1.2 mg/dL	
ALT	33	7-56 U/L	
AST	25	10-40 U/L	
Alkphos	70	44-147 U/L	
Lactic acid	0.9	0.5-2.2 mmol/L	
ESR auto	110 (H)	0-20 mm/h (males), 0-30 mm/h (females)	

**Table 3:** Urinalysis obtained on postoperative day 10 UA: urinalysis

Lab name	Lab value		Reference range
Color	Amber	Yellow (light/pale to dark/deep amber)	
Appearance	Clear	Clear	
Specific gravity	≤1.005	1.005-1.030	
UA pH	5.5	4.6-8.0	
UA glucose	Negative	Negative	
UA bilirubin	Negative	Negative	
UA ketones	Negative	Negative	
Blood	Small (A)	Negative	
Protein	30 (A)	Negative or trace amounts	
Urobilinogen	0.2	0.1-1.0 mg/dL	
Nitrite	Negative	Negative	
Leukocyte esterase	Negative	Negative	

**Table 4:** Blood gasses obtained on postoperative day 10 ven: venous

Lab name	Lab value		Reference range
Ph Bld gas, ven	7.36	7.31-7.41	
PpCO_2_ ven	45	41-51 mmHg	
PO_2_, ven	25 (L)	30-50 mmHg	
HCO_3_ ven	25.4	22-28 mEq/L	
BE ven	0	-2 to +3 mEq/L	
SO_2_ calc ven	41 (L)	60-80%	
Lactic acid ven	0.69	0.5-2.2 mmol/L	
TCO_2_, calc, ven	27	23-29 mEq/L	

**Figure 1:**
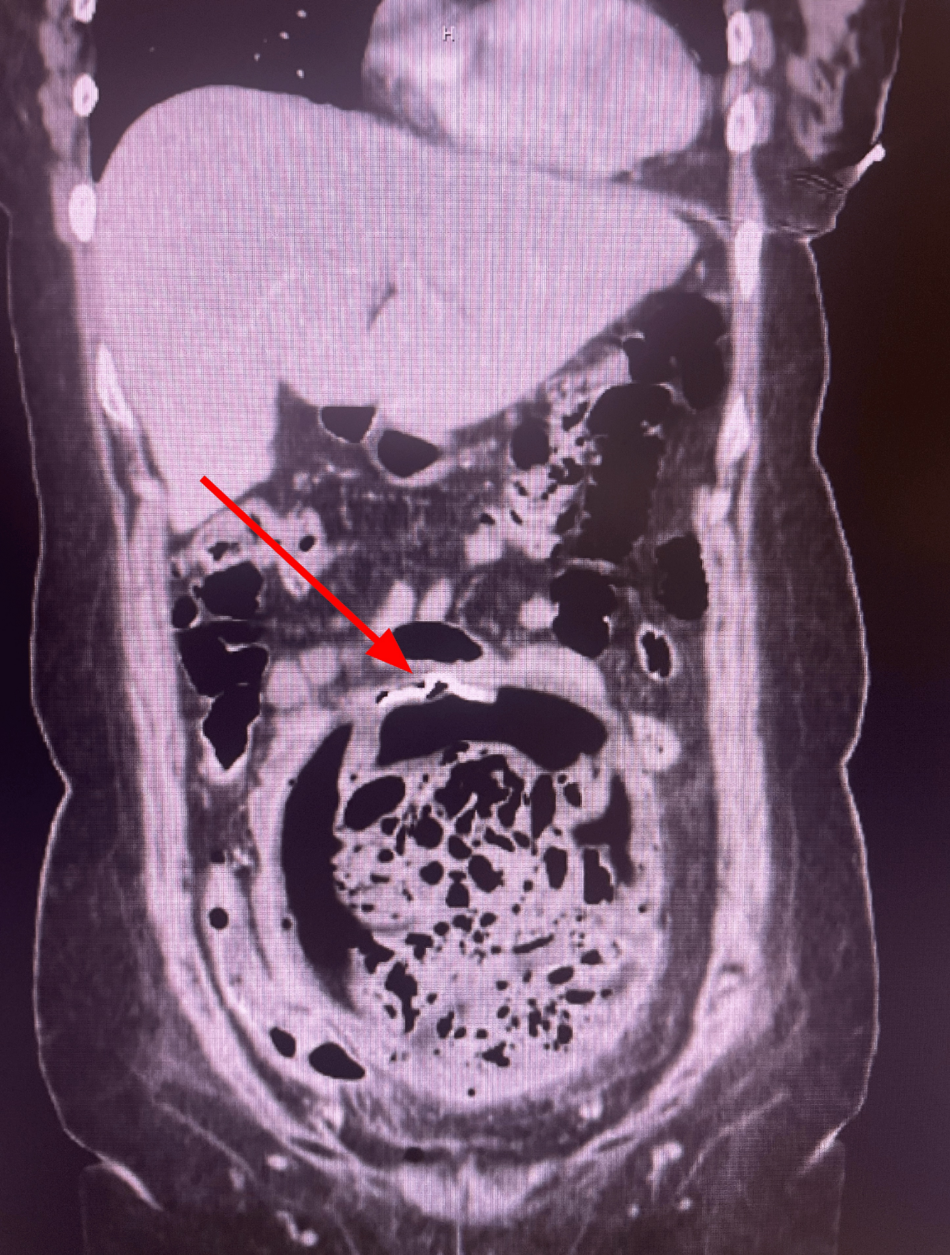
Coronal CT reveals a large, complex intra-abdominal abscess measuring approximately 9.5 x 15 x 16 cm, located in the mid-abdomen/pelvis region. A radiodense ribbon consistent with a retained surgical towel is visible within the abscess cavity, indicated by the red arrow

**Figure 2:**
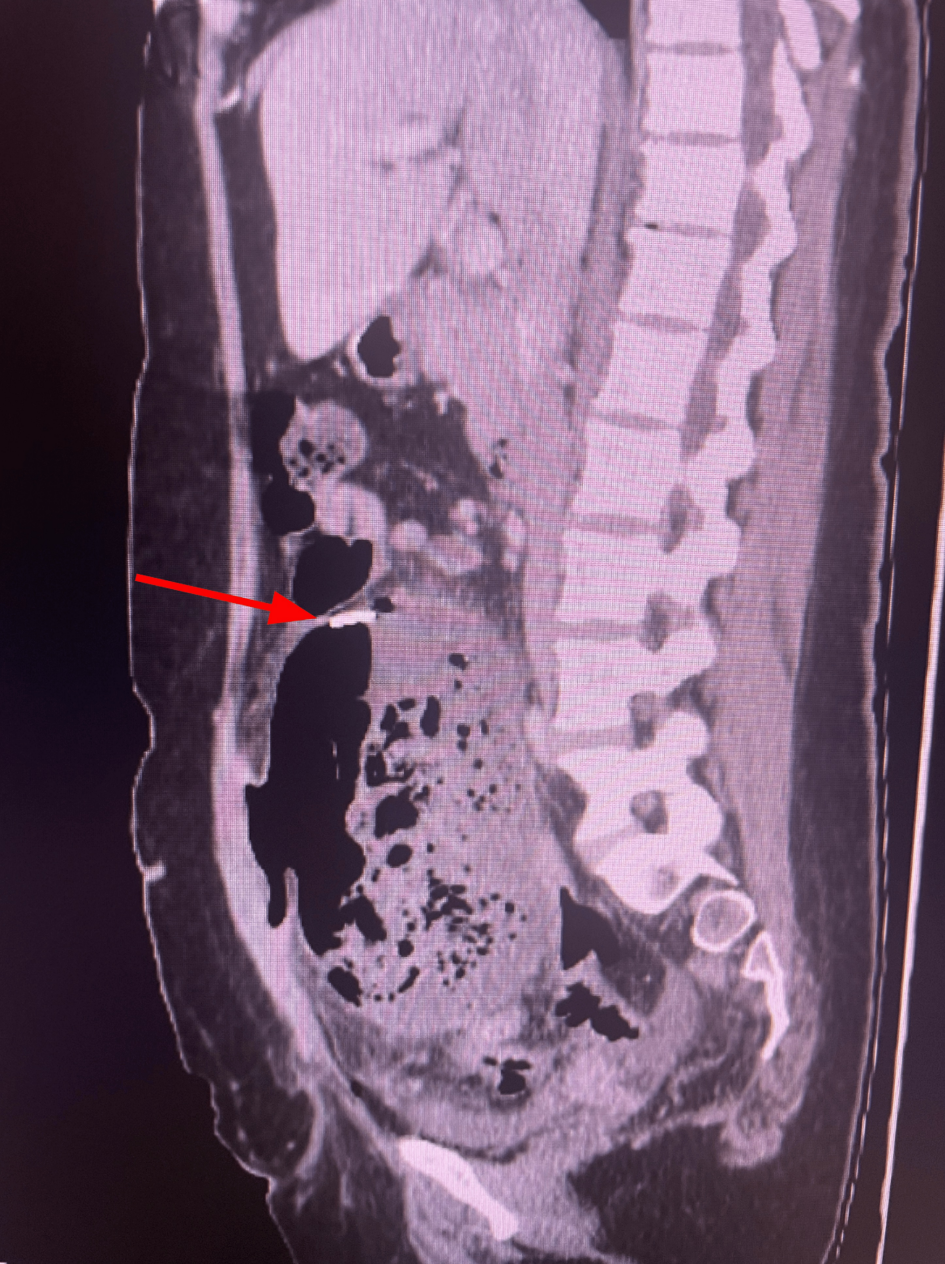
Sagittal CT shows a large intra-abdominal abscess in the lower abdomen and pelvis. A radiodense ribbon, indicated by the red arrow, is seen within the abscess cavity, consistent with a retained surgical towel

**Figure 3:**
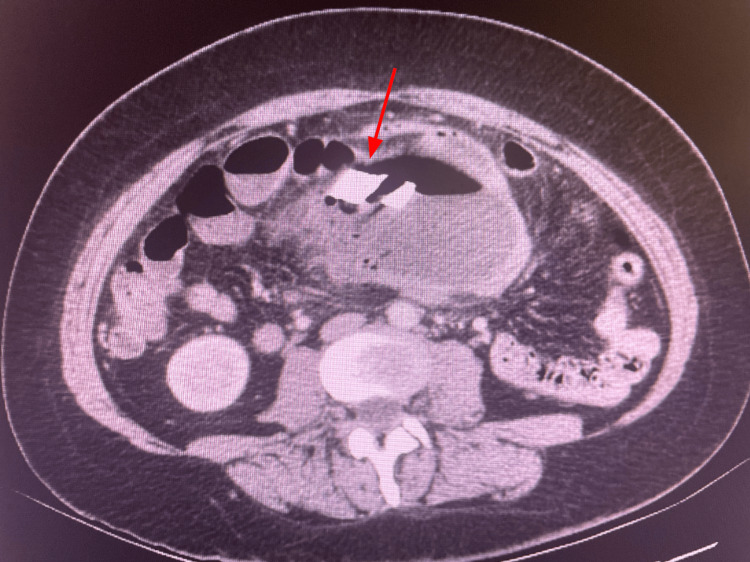
Transverse CT shows a large intra-abdominal abscess in the lower abdomen and pelvis. A radiodense ribbon, indicated by the red arrow, is seen within the abscess cavity, consistent with a retained surgical towel

Abdominal and pelvic CT scans were ordered to assess for any postoperative complications. The abdominal CT (Figures 1-3) revealed an unexpected and alarming finding: the radiologist described “a large complex measuring 9.5 x 15 x 16 cm with a radiodense ribbon near the superior aspect in the mid-abdomen/pelvis region.” The presence of an apparent retained surgical item within the abdominal cavity and the formation of a massive abscess surrounding it were confirmed. The patient was promptly admitted for emergent surgical intervention.

The surgeon proceeded with a laparotomy incision, followed down through the fascia, and immediately encountered purulent material, which was removed with suction. A retained white surgical towel was encountered within the abscess cavity, which was carefully extracted and subsequently handed off for pathological inspection. Multiple adhesions of the bowel to the abscess were noted, which consisted of omentum, retroperitoneum, mesentery, and the wall of the small bowel. The abscess caused by the foreign body had eroded partially into the serosa of the small bowel, necessitating suture repair.

Microbiological analysis of the abscess identified a multidrug-resistant organism with characteristics of extended-spectrum beta-lactamase production, resistance to penicillins, aztreonam, and first, second, and third-generation cephalosporins. Gram-positive cocci were also moderately identified, indicating a complex microbial composition. Retained surgical items are a rare but severe complication of surgery, with potentially devastating consequences for patients. In this case, a surgical towel was inadvertently left within the patient's abdomen during the hysterectomy performed in Mexico. The retained foreign body led to the formation of a massive abscess, causing the patient's symptoms of pain, fever, and malaise.

The management of retained surgical items typically involves radiographic imaging, surgical exploration, and removal of the foreign body, followed by thorough irrigation and drainage of any associated abscess. Antibiotics are administered to address the infection. The patient was treated with intravenous vancomycin, piperacillin, and tazobactam. A nasogastric tube and Jackson-Pratt drain were placed postoperatively for drainage. Postoperative radiographic studies confirmed the resolution of the infection, absence of free air, foreign bodies, and other abnormalities after the surgical removal of the foreign object.

## Discussion

This case underscores the importance of effective communication between patients and healthcare providers, particularly when alarming postoperative symptoms are involved. A multidisciplinary approach required in managing complications arising from retained foreign bodies is shown in this case; appropriate antibiotic management, pain control adjustments, and drainage interventions illustrate the importance of careful, intentional surgical techniques and postoperative assessments and management. This case also highlights the potentially severe consequences of nosocomial infections with highly transmissible and multi-drug-resistant organisms, showing the need for frequent monitoring and appropriate antimicrobial treatment.

In comparing the prevalence of infections leading to abdominal abscesses in Mexico and the United States, it is essential to consider various factors, including healthcare infrastructure, hygiene practices, and surgical standards. While comprehensive data directly comparing these rates is scarce, existing literature provides insights into the general infectious disease landscape in both countries. In Mexico, the incidence of healthcare-associated infections (HAIs) has been a concern; studies have reported variable rates of HAIs, emphasizing challenges in infection prevention and control practices within healthcare facilities [2]. These infections can contribute to the development of complications such as abdominal abscesses. Mexico faces specific challenges in preventing surgical site infections, which may include limited resources, variable adherence to aseptic practices, and infrastructure disparities [3]. The emergence of multi-drug-resistant organisms in Mexico has also been documented [4]. Challenges related to antibiotic stewardship and access to appropriate healthcare may contribute to the persistence of resistant strains and increase the risk of infections and abscess formation [4].

In this case, it is important to note that the patient contacted her surgeon in Mexico when postoperative symptoms worsened. However, the patient was informed that the symptoms were not out of the ordinary, proven inaccurate by the following events. This miscommunication underscores the significance of accurate and detailed communication between patients and healthcare providers and the need for a timely and appropriate response from healthcare staff based on evolving clinical scenarios [5]. Such instances of misguidance can contribute to delayed diagnosis and treatment, potentially leading to complications such as the development of abdominal abscesses.

The inadvertent retention of a surgical towel in the patient's case shows the importance of stringent surgical safety measures. Studies have suggested that lapses in safety protocols may contribute to the occurrence of retained foreign bodies and subsequent infections [6]. While direct comparative studies are limited, the United States has invested significantly in infection prevention strategies, surveillance systems, and stringent surgical protocols. Implementing these measures has contributed to comparatively lower rates of HAIs, including abdominal abscesses, in the United States healthcare settings [7].

Enhancing surgical safety requires a culture of accountability and adherence to best practices, which includes fostering an environment where team members feel empowered to speak up about concerns during procedures and implementing checklists. Additionally, managing complications from retained foreign bodies necessitates a collaborative approach involving surgeons, infectious disease specialists, and nursing staff, with protocols for regular case reviews and communication enhancing overall patient care.

Healthcare-associated challenges, antibiotic resistance, and communication gaps may influence the prevalence of infections leading to abdominal abscesses in Mexico. Comparatively, the United States, with increased healthcare infrastructure and more cautious infection prevention protocols, demonstrates a lower incidence of such complications. However, more comprehensive studies are needed to better understand the factors contributing to abdominal abscesses in both contexts.

## Conclusions

This unfortunate patient incident, as highlighted in this case report, highlights the necessity of prioritizing patient safety in surgical care through strict protocol adherence before, during, and after surgery. This patient experienced the postoperative severe complication of a retained surgical towel following a hysterectomy in Mexico, demonstrating the increased potential risks and harm inherent in medical tourism. The foreign body led to a sizable intra-abdominal abscess. It imposed an unnecessary burden on the patient, further complicated by the inability to realistically follow up in person with the surgeon due to geographical distance and travel costs. This case presents an opportunity to reinforce adherence to surgical safety protocols, including safety checklists, instrument counts, and high-quality follow-up care. It also highlights the likely challenges of quality of care in medical travel. It is a caution for those seeking medical care abroad and a call to action for healthcare providers to maintain the highest standards of surgical care and patient follow-up. In presenting this case, we hope to contribute to ongoing reforms that advocate for surgical safety and reduce adverse and sentinel events, emphasizing quality patient-centered care regardless of geographical boundaries.
